# SA Rugby Injury and Illness Surveillance and Prevention Project (SARIISPP): The Currie Cup Premiership Competition Injury Surveillance Report, 2022

**DOI:** 10.17159/2078-516X/2023/v35i1a16880

**Published:** 2023-11-16

## Executive Summary

As part of the South African Rugby Injury and Illness Surveillance and Prevention Project (SARIISPP), the medical doctors and medical support staff of the respective teams recorded the injury data for the annual Currie Cup 2022 Premiership Division Competition (‘Currie Cup’). SARIISPP has been collecting and analysing these data annually since 2014 for the Currie Cup tournament. All seven teams are required to record the injuries that occur in each match and training session in the team throughout the season. The strength and conditioning coaches also recorded their training sessions for training exposure data throughout the season. By combining match exposure data, training exposure data and injury data, SARIISPP aims to obtain a more complete understanding of the factors contributing to player injury risk.

The analysis reveals injury patterns and allows comparison across different years, tournaments, teams, the scientific literature, and internationally. During this investigation, areas of concern are identified and appropriate changes in the game, tournament structure, or medical support services are considered based on the evidence. Moreover, injury-specific interventions can be developed and implemented when the evidence supports such actions.

This report uses injury burden and injury rate as key metrics for analysis. It is worth noting that even if teams maintain a low injury rate, injuries of high severity still impose a substantial burden on the team. Such injuries lead to a significant number of training and match days lost due to injury. This highlights the importance of collecting data on injury severity too rather than relying solely on injury rates.

The injury rates are expressed as the mean (95% confidence interval) per 1000 player hours. The injury rate of Time-Loss injuries for the Currie Cup 2022 was 69 (57 to 81) injuries per 1000 player hours; the lowest injury rate recorded since 2015. This is however not significantly different to the international meta-analysis injury rate of 91 (77 to 106) injuries per 1000 player hours [1], and falls within the expected limits of season-to-season variation for the Currie Cup. This equates to 1.4 injuries per team per match, with an injury burden of 2139 days lost per 1000 player hours.

The Cell C Sharks had the highest injury rate for Time-loss injuries throughout the Currie Cup 2022 tournament. DHL Western Province had a significantly lower injury rate in 2022 compared to their 2014–2021 tournament average. The Vodacom Blue Bulls had the highest average severity of 50 days absent per injury, whereas the Airlink Pumas had the lowest average severity of 13 days absent per injury. The Airlink Pumas, who subsequently won the tournament, also had the lowest injury rate compared to the other teams in the tournament. The average severity of Time-Loss injuries in the 2022 tournament was 31 days, which is marginally higher than the 27 days reported in the international meta-analysis [1]. The median injury severity of all Time-Loss injuries was 19 days, with 25% of injuries lasting 10 days or less and 25% of injuries lasting 44 days or more due to injury.

The most common injury type observed during the 2022 Currie Cup tournament was ligament sprain, followed by muscle (rupture/strain/tear) injuries and central nervous system injuries, which ranked second and third, respectively.

The shoulder, head, and ankle were the most injured body locations. Shoulder injuries increased by six per cent since the 2021 season. The number and incidence of concussions also increased in the 2022 Currie Cup tournament with an injury incidence of 10 (5 to 14) injuries per 1000 player hours. *Being Tackled* accounted for the highest number of injuries in the 2022 Currie Cup tournament followed by *Open Play* and *Tackling.*

A total of 61 Time-Loss training injuries were sustained in the Currie Cup 2022. Among all the Time-Loss injuries incurred, 34% occurred during training. This equates to an incidence of 2 (1.5 to 2.5) injuries per 1000 training hours which is lower than the injury incidence of 3 (2 to 4) injuries per 1000 training hours reported in the meta-analysis [1]. Time-Loss training injuries resulted in an average severity of 34 days absent.[Fig f22-2078-516x-35-v35i1a16880]

**Figure f22-2078-516x-35-v35i1a16880:**
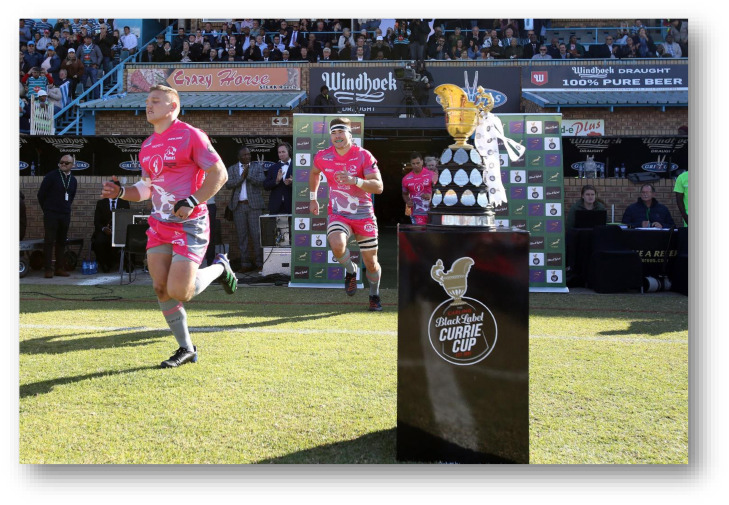


## Introduction

In 2014, the South African Rugby Union (SA Rugby) introduced a standardised injury surveillance format for the Currie Cup Premiership Division Competition as part of the SA Rugby Injury and Illness Surveillance and Prevention Project (SARIISPP). This format required the team’s medical doctor or medical support staff to record all relevant injury data from matches and training sessions using the standardised *BokSmart* injury surveillance data capture format. The definitions and reporting format used in this system are aligned with the recent IOC consensus statement for injury recording in sport [2], and with the consensus statement on injury definitions and data collection procedures for studies of injuries in rugby union [3].

Injury surveillance is an essential step in injury prevention. Specifically, injury surveillance is important for developing injury prevention strategies, and assessing their efficacy and effectiveness after implementation. By capturing injury surveillance in a standardized format, it becomes possible to compare injury rates between teams participating in the same tournament, to track tournament injuries over consecutive years, and compare findings with other rugby injury surveillance studies. This standardised approach enables comprehensive analysis and enhances the ability to make well-informed evidence-based decisions regarding injury patterns and potential prevention strategies.

Reports on rugby tournament injuries typically present the injury numbers as a rate (or incidence) i.e., the total number of injuries divided by the total amount of time exposed to the risk of experiencing an injury. The standardised format is to present the number of injuries per 1000 player exposure hours. Match exposure hours are calculated as the number of matches played multiplied by the number of exposed players (30) and the match duration (80 mins); for team-specific match-related exposure 15 players would be utilised. Training exposure hours are calculated as the average number of players present at training multiplied by the average time spent training each week. These values are then summed to obtain the training exposure hours over the competition period. In this report the standardised injury rates have been provided to allow for comparison with other reports. Every effort has been made to present these rates on a ‘per team’ and ‘per match’ basis for easier and more practical interpretation.

Since 2016, the Currie Cup medical doctors and medical support staff were asked to record the physical return to play date of the injured players, thereby allowing for the actual severity of the injury to be calculated. Injury burden is a combination of the injury rate and severity and is expressed as the number of days absent from training and matches per 1000 player hours. Throughout this report, only actual, rather than predicted severity is used for analysis.

The report includes data from the 2014 and 2015 seasons only in those sections that report on injury numbers and incidence. The sections that report on injury severity and burden begin with the 2016 season, which was the first-time actual severity data was collected.

In the Currie Cup 2020/21 seasonal report, the South African Rugby Injury and Illness Surveillance and Prevention Project (SARIISPP) began capturing Time-Loss training injuries and training exposure data. This addition enables SARIISPP to gain a more comprehensive understanding of injury data by combining match and training exposure and injury data.

An inherent bias with most injury surveillance studies is that the teams’ medical doctors or medical support staff are responsible for entering their team’s injury data. As no audit process is done on the collection of these data, in many cases, the accuracy of the data depends on the compliance of the medical doctors or medical support staff. This potential limitation is present in most injury surveillance studies. To minimise this potential limitation, SARIISPP had a project coordinator who maintained regular communication with the medical doctors or medical support staff. This ensured that data capturing was up to date.

The Currie Cup 2022 semi-finals were contested between Airlink Pumas vs. Toyota Cheetahs and Vodacom Blue Bulls vs. Windhoek Draught Griquas. The final was between Airlink Pumas vs. Windhoek Draught Griquas, with the Airlink Pumas eventually winning the tournament for the first time in history.[Fig f23-2078-516x-35-v35i1a16880]

**Figure f23-2078-516x-35-v35i1a16880:**
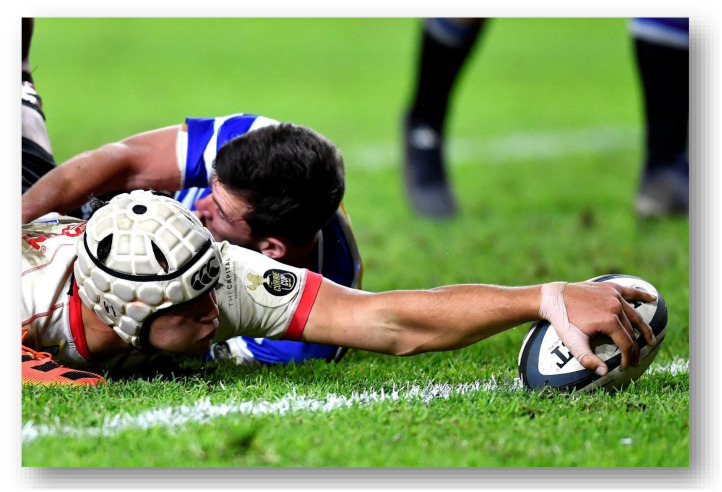


## Definitions

All definitions are originally based on the 2007 consensus statement for injury reporting in rugby union [3] and have since been realigned with the latest International Olympic Committee (IOC) consensus statement for methods of recording and reporting epidemiological data on injury and illness in sport [2].

### MEDICAL ATTENTION INJURY

All injuries that were seen by the teams’ medical doctor or medical support staff were classified as Medical Attention injuries. These injuries are defined by the 2007 statement as an “*injury that results in a player receiving medical attention”* [3], and by the more recent IOC statement as *“a health problem that results in an athlete receiving medical attention”* [2]. For clarity, this means an injury sustained by a rugby union player during a match or training session that prevented or would have prevented the player from taking full part in all rugby training activities and/or match play for more than 1 day following the day of injury, irrespective of whether match or training sessions were scheduled [4].

### TIME-LOSS INJURY

Medical Attention injuries were further categorised as Time-Loss injuries, where appropriate, and defined by the 2007 statement as, “*an injury that results in a player being unable to take a full part in future rugby training or match play*” [3]. The IOC definition is, *“a health problem that results in a player being unable to complete the current or future training session or competition”* [2].

### INJURY RATE

For this report, an injury rate is defined as the number of injuries expressed per 1000 player exposure hours. This method of expressing injury rate has been used in previous years’ reports of the Currie Cup Premiership tournament and other international literature, and therefore makes comparisons easy. Moreover, the injury rate is expressed as a mean with 95% confidence intervals. A 95% confidence interval around a mean value indicates that there is a 95% chance (i.e., very high chance) that the true value falls within this range. In this report, we present the 95% confidence intervals assuming normal distribution of the data and use the approach of examining the overlap of the confidence intervals, to determine whether the injury incidences are significantly different; if the range of confidence interval values of two comparisons do not overlap, there is a strong chance (95%) that their injury rates are different from each other. We have opted for this method because it is easy to use, conservative and less likely to produce false positive results [5].

### MEDIAN (INTERQUARTILE RANGE)

When numbers are ordered from the lowest to highest, the median is the value which separates the higher half of the values from the lower half of the values. Simply put, it is the middle value of a list of ranked numbers. The interquartile range (IQR) describes the spread of the data. When rank-ordered data are divided into quartiles the first and the third quartile represents the value under which 25% and 75% of the data points fall, respectively. As an example, consider a team with a median injury severity of 32 days (IQR 7 to 40). This means that when the teams’ injury severities are ranked in order the mid-point or median of the injury severities is 32 days. Also 25% of their injuries result in 7 or less days absent from training and matches and 25% of their injuries result in 40 days or more absent from training and matches.

### NEW, SUBSEQUENT AND RECURRENT INJURIES

In 2022, in the Currie Cup Premiership Division Competition, a ‘*New Injury’* was defined as when a player sustained his first injury. Any injury the *same* player sustained after this initial injury was defined as a *‘Subsequent Injury*.

According to the IOC statement, any subsequent injury to the same site and of the same type is referred to as a ‘*Recurrence’* if the index injury was fully recovered before reinjury, and as an *‘Exacerbation’* if the index injury was not yet fully recovered [2].

To provide more detail on the subsequent injuries for practitioners, we have further categorized the subsequent injuries in this report into one of four groups based on the Orchard Sports Injury and Illness Classification System (OSIICS) classification diagnosis:

- Different site - Different type
- Different site - Same type
- Same site - Different type
- Same site - Same type

According to the 2007 Consensus Statement for rugby, any subsequent injury classified as ‘Same site - Same type’ was a *‘Recurrent injury’* [3].

### INJURY SEVERITY

The total severity of an injury is defined as *“the number of days that have elapsed from the date of injury to the date of the player’s return to full participation in team training and availability for match selection”* [2,3].The actual severity of each injury is classified by the severity groupings provided in the 2007 consensus statement; *Slight* (0–1 days lost), *Minimal* (2–3 days lost), *Mild* (4–7 days lost), *Moderate* (8–28 days lost), *Severe* (>28 days lost), *Career ending* and *Non-fatal catastrophic* [3]. To align with the latest IOC statement the injuries have been re-grouped to reflect the severity groupings *‘1–7 days’, ‘8–28 days’ and ‘>28 days’* [*2*].

The average severity represents the average number of days lost per injury when dividing the accumulated total number of days lost by the total number of injury events. For example, a team may have a total severity of 550 days absent, accumulated from 22 injuries. The average severity of the team’s injuries would therefore be 550/22, which equals, on average 25 days absent per injury.

### INJURY BURDEN

Injury burden is determined by the combination of injury rate and severity. It is calculated by multiplying the injury rate by the average severity (number of days lost due to injury) and is expressed as the number of days absent per 1000 player hours. For example, consider a team with an injury rate of 75 injuries per 1000 player exposure hours, and an average severity of 38 days lost per injury. In this case, the injury burden for the team would be calculated as 2850 days absent per 1000 player hours (i.e., 75 x 38 = 2850).

### OPERATIONAL INJURY BURDEN

The operational burden is the expected number of days lost per injury per team for every match played over the tournament or season. The measure is an extrapolation of injury rates and severities over a season and includes the most severe injuries together with the least severe injuries in its estimation. For example, if a team has an operational injury burden of 2 days, it means that based on their injury rates and average severity, on average, 2 days absence can be expected from every match injury the team sustains.

### META-ANALYSIS

A meta-analysis is a study using statistical methods to combine multiple scientific studies with varying levels of evidence on the same topic. The goal is to determine overall defining patterns and results based on the combined data. As such, it represents the highest level of scientific evidence available. The findings in this report are compared to the data in the most recent meta-analysis, which was published in 2021. The meta-analysis specifically focuses on rugby union injuries at an elite professional level [1].[Fig f24-2078-516x-35-v35i1a16880]

**Figure f24-2078-516x-35-v35i1a16880:**
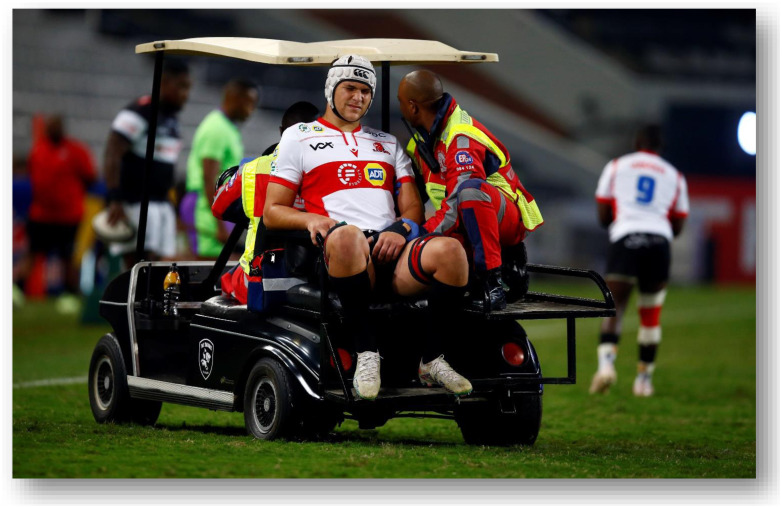


## MATCH INJURIES

### Injured players

During the Currie Cup 2022, 94 players sustained a total of 121 Time-Loss injuries. Due to squad changes over the tournament duration for various reasons, a total number of 330 different players were physically exposed to injury at some point while playing rugby matches as part of the Currie Cup 2022 tournament. However, for analysis and exposure calculation purposes, we assumed a total of 161 players were available for playing rugby on match days in the tournament (7 teams x 23 players per match-day squad). Fifty-eight percent (58%) of the 161 available match-day players sustained a match injury during the tournament (Figure 1a). The proportion of players who sustained one Time-loss injury increased slightly from 2021 to 2022. Furthermore, the proportion of players who experienced 3 or 4 injuries decreased similarly from 2021 to 2022 (Figure 1b). Only the absolute number of Time-loss injuries were analysed further in this report (n = 121), regardless of the number of players who sustained them.

### Overall Injury Rate

Only Time-loss injuries have been analysed in this report because these injuries are more comparable between different teams, tournaments and with the published scientific literature [1]. As mentioned previously, Time-loss injuries resulted in players missing a match or training session.

**Figure 2:**
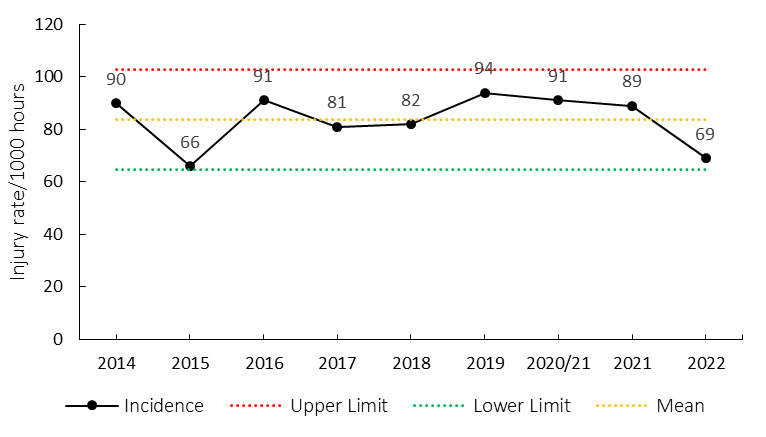
Injury incidence of Time-Loss match injuries over the surveillance period with mean ± standard deviations shown. The red dotted line represents the mean plus standard deviation. The green dotted line represents the mean minus standard deviation. 2020/21 – was a hybrid tournament structure that started in 2020 and carried over into the beginning of the 2021 season due to Covid-19 lockdown interruptions.

The overall match injury incidence for the Currie Cup 2022 was 69 (57 to 81) injuries per 1000 player exposure hours: the lowest injury rate since 2015. The 2022 Currie Cup tournament’s injury rate is however not significantly different to the international meta-analysis injury rate of 91 (77 to 106) injuries per 1000 player hours [1] and falls within the season-to-season variation for the Currie Cup, based on the last 8 years’ collective data (Figure 2). An injury incidence of 69 injuries per 1000 player hours equates to 1.4 injuries per team per match.

**Figure 3:**
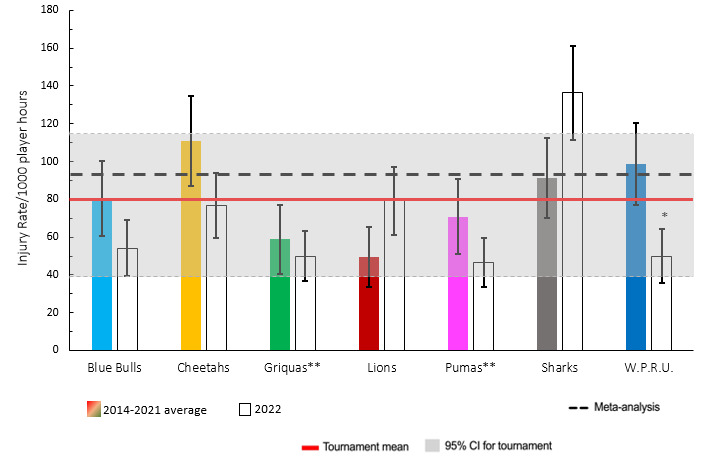
Injury incidence rates for Time-Loss injuries experienced by each team in the Currie Cup 2022 in comparison to their 2014–2021 averaged injury rate. (**) Average injury rates for Pumas 2015 – 2021 and Griquas for 2015, 2016, 2018, 2019, 2020/21 and 2021. Asterisk (*) indicates that a team’s 2022 injury rate is significantly different to their 2014–2021 averaged injury rate. The whisker lines for each bar represents the 95% Confidence Interval.

When comparing the team’s 2014–2021 averaged tournament injury incidence to their 2022 season’s injury incidence data, the DHL Western Province experienced a significantly lower injury incidence rate in 2022 (Figure 3). The Sharks had a significantly higher injury rate compared to all other teams in 2022. No team showed significantly higher injury incidences than their 2014–2021 tournament averages.

Overall, the combined average injury incidence of 80 (39 to 115) injuries per 1000 player hours for all the teams over the last 9 years is similar to the international meta-analysis summary of 91 (77 to 106) injuries per 1000 player hours [1].

### Injury incidence over the season

**Figure 4:**
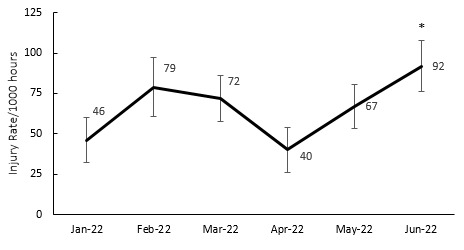
Match injury incidence per month of the 2022 Currie Cup season. Asterisk (*) indicates that the injury incidence is significantly higher in June than in January and April. The whiskers for each point represent the 95% Confidence Intervals.

The 2022 Currie Cup tournament consisted of two rounds of matches and took place from January to June. Unlike previous years, when the competition was held in the second half of the year and either had one or two rounds, this year’s format was changed to a double round competition in the first half of the year. When examining the Time-loss injury incidence during the 2022 Currie Cup tournament, it was found that the injury incidence in June was significantly higher compared to January and April. Throughout the other months in the 2022 season, there were no significant differences (Figure 4).

### Overall Severity

**Figure 5:**
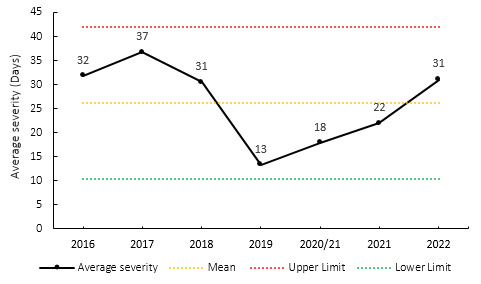
Mean severity of Time-Loss match injuries over the surveillance period with mean ± standard deviations shown. The red dotted line represents the mean plus standard deviation. The green dotted line represents the mean minus standard deviation. 2020/21 – was a hybrid tournament structure that started in 2020 and carried over into the beginning of the 2021 season due to Covid-19 lockdown interruptions.

The average severity of match injuries for the Currie Cup 2022 was 31 days, which is higher than the averaged severities for the Currie Cup tournament 2016–2021 (25 days) but was within the expected season-to-season variation (Figure 5). There has been a consistent increase in match severity since 2019, although it has not exceeded the level observed between 2016 and 2018. The median severity in 2022 was 19 days (IQR 10 to 44). This means that the half-way mark of the injury severities was 19 days, with 25% of all Time-Loss injuries lasting 10 days or less and 25% lasting 44 days or longer.

When the medical doctors or medical support staff clinically assessed the injured player, they recorded the injury time from the date the injury occurred, as the starting date. Similarly, when the player returned to full participation in team training and availability for match selection, the return to play date was recorded. The injury severity was determined from the difference between these two dates.

These data are grouped to align with the latest IOC statement. The severity groupings include *‘1–7 days’, ‘8–28 days’ and ‘>28 days’* [*2*].[Fig f25-2078-516x-35-v35i1a16880]

**Figure f25-2078-516x-35-v35i1a16880:**
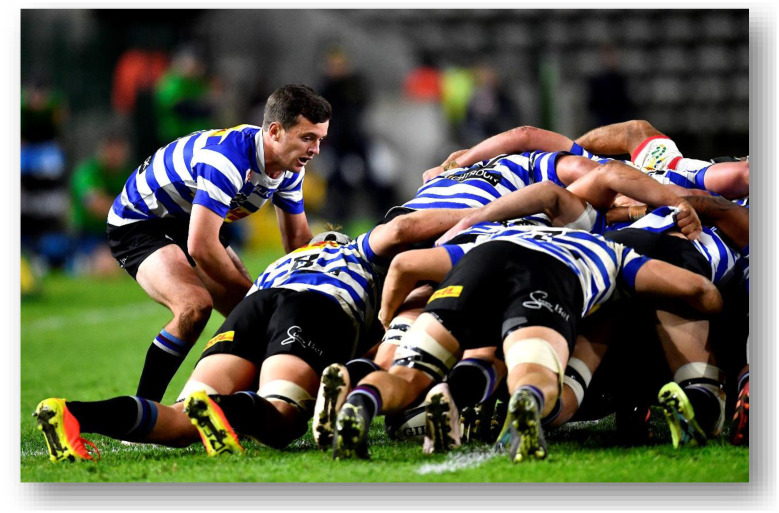


**Figure 6:**
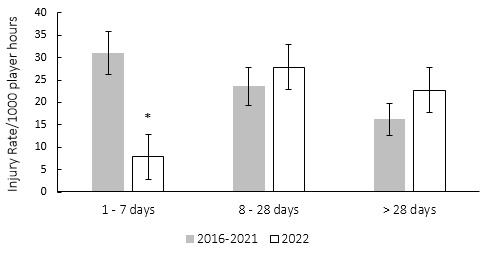
The actual severity category injury rates for the Currie Cup 2022 in comparison to the averaged injury rates for the 2016–2021 actual severity categories. The whiskers for each bar represent the 95% Confidence Intervals.

Figure 6 compares the injury severity rates for the Currie Cup 2022 tournament to the averaged injury severity rates of the 2016–2021 tournaments. Injury rates in the severity category of ‘*1–7 days’* were significantly lower in 2022 compared to their 2016–2021 average (Figure 6). Interestingly, the injury severity categories in 2022 show a trend towards more severe injuries as opposed to the trend demonstrated by the 2016–2021 accumulated data, which has a trend towards the less severe injuries.[Fig f26-2078-516x-35-v35i1a16880]

**Figure f26-2078-516x-35-v35i1a16880:**
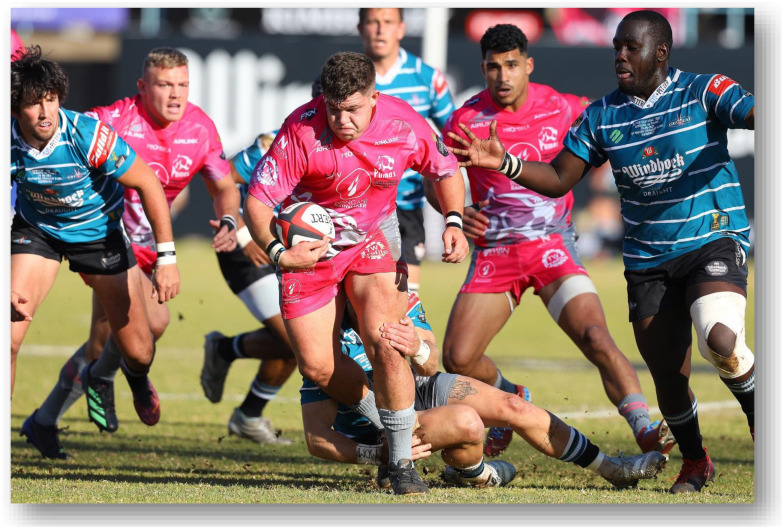


Table 1 describes the actual severity of each teams’ Time-Loss injuries for the Currie Cup 2022. The Airlink Pumas have been used as a worked example to explain Table 1. The Airlink Pumas sustained 0.9 injuries per match, meaning that for every 1.1 matches played, they sustained one injury. The Airlink Pumas lost 163 training and match days due to injury. This equates to an average of 13 training and match days lost for every injury sustained. The burden of the team’s injuries equates to 582 days lost per 1000 player hours. Translating this to an operational burden per match shows that the Airlink Pumas lost 11.6 days per injury per match over the season. The median injury severity for the Airlink Pumas was 11 days (IQR 10 to 27). This means that when severities of the Airlink Pumas Time-Loss injuries were rank ordered, the midpoint of the severities was 11 days off from rugby, with 25% of their injuries lasting equal to or less than 10 days off and 25% of their injuries lasting equal to or longer than 27 days off.

**Figure 7:**
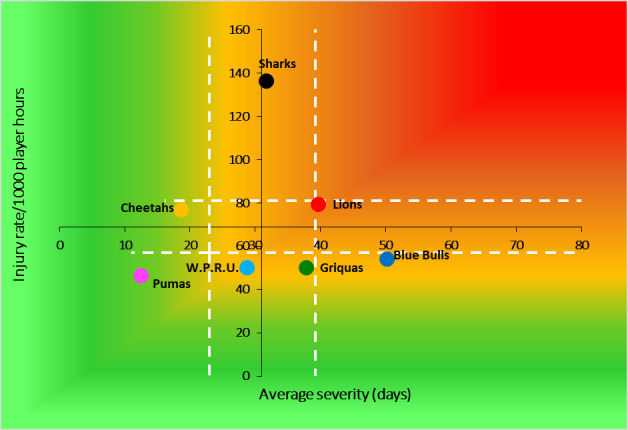
Injury rate plotted against the average severity of Time-Loss injuries for each participating team in the Currie Cup 2022. The Y-axis Average Injury Rate is expressed as the tournament average (the vertical white dotted lines represent 95% Confidence Intervals) and X-axis Average Severity is expressed as the average (the horizontal white dotted lines represent 95% Confidence Intervals) of all the individual injury severities in the tournament.

The Cell C Sharks had the highest Time-Loss injury rate by quite some distance, followed by the Sigma Golden Lions and then Toyota Free State Cheetahs. In contrast, the Airlink Pumas had the lowest injury rate, the lowest severity, and by extension the lowest injury burden per team (Table 1; Figure 7). It has been shown in previous studies that teams with lower injury rates had more success in the Currie Cup competition [6, 7]. It has also been shown that injury burden needs to be considered for success and not simply injury rates alone [8]. Teams who fall in the green zone (below average and 95%CI), will generally not be impacted as much by their injury burden, regardless of whether their injury rate or average severity is relatively high. As soon as the combination of rate and severity moves into the orange (close to average) and/or red zone (above average and 95% CI), the impact on team performance and player availability becomes more problematic. None of the teams participating in the 2022 Currie Cup were in the red zones. However, Cell C Sharks showed the highest injury burden because of their combination of high injury rates and severity. This was followed by the Sigma Golden Lions.

All the data in this report are aligned with the 2019 IOC consensus statement [2] and are further presented as such to compare against previous season reports and the international meta-analysis [1]. Table 2 presents the Currie Cup 2022 injury data in the format recommended by the 2019 IOC consensus statement. This table provides an overview of the Tissue and Pathology types of injuries sustained during the 2022 season. This format is used throughout this report.

### New, Subsequent and Recurrent Injuries

During the Currie Cup 2022, the overall injury incidence for *New injuries* was 53 (43 to 64) injuries per 1000 player hours. This is a similar injury rate to the Currie Cup 2021.

**Figure 8:**
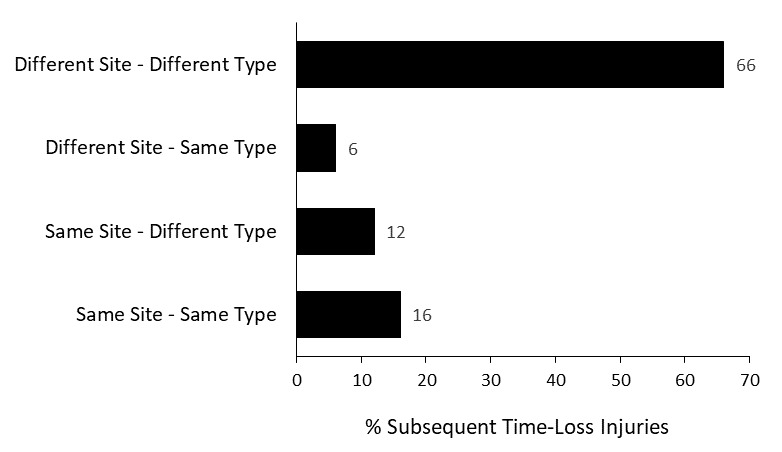
Classification of subsequent injuries for the Currie Cup 2022. Data expressed as a % of subsequent Time-Loss injuries.

Seventy-one players experienced only one injury during the Currie Cup 2022 season (76% of all injured players). Sixty-six percent (66%) of subsequent injuries to those 23 players sustaining multiple injury events during the season (Figure 1a), occurred at a different anatomical site and were of a different type when compared to the initial index injury. ‘*Different site – different type*’, ‘*different site – same type*’ and ‘*same site – different type*’ are classified as subsequent new injuries. Figure 8 shows the percentage subsequent Time-loss injuries.[Fig f27-2078-516x-35-v35i1a16880]

**Figure f27-2078-516x-35-v35i1a16880:**
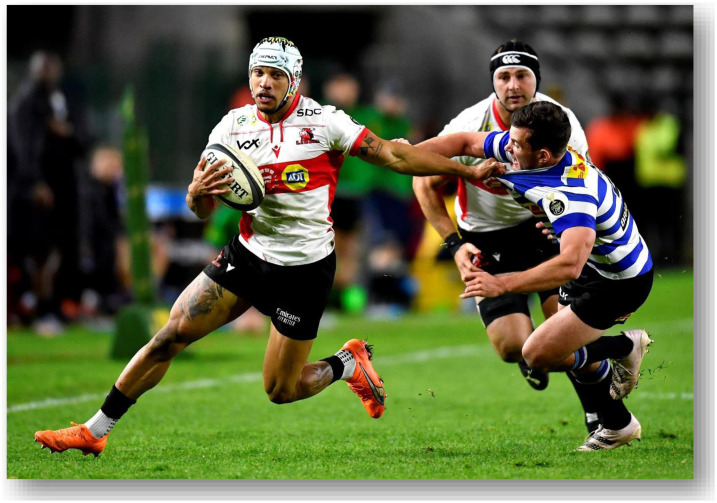


A subsequent *recurrent* injury was any subsequent injury classified as ‘*same site – same type*’, which refers to the same location and same tissue type involved as the original index injury. Only four subsequent recurrent injuries occurred in the Currie Cup 2022.

The injury incidence in 2022 for subsequent recurrent injuries was 3 (0 to 5) injuries per 1000 player hours, which is slightly lower than the 2021 tournament’s injury incidence of 4 (1 to 7) injuries per 1000 player hours.

There is a slight increase in the proportion of new injuries and a corresponding decrease in subsequent *recurrent* injuries compared to the Currie Cup 2021 tournament (Table 3).

### Injury Type

Overall, Ligament sprain was the most common Time-loss injury during the Currie Cup 2022 (26%), followed by Muscle (rupture/strain/tear) injuries (21%).

The median severity for Ligament sprain injuries was 24 days with 25% of injuries resulting in 13 or less days absent from training and matches, and 25% of injuries resulting in 75 or more days absent from training and matches *(*Table 4). The average severity was 42 days absent.

**Figure 9:**
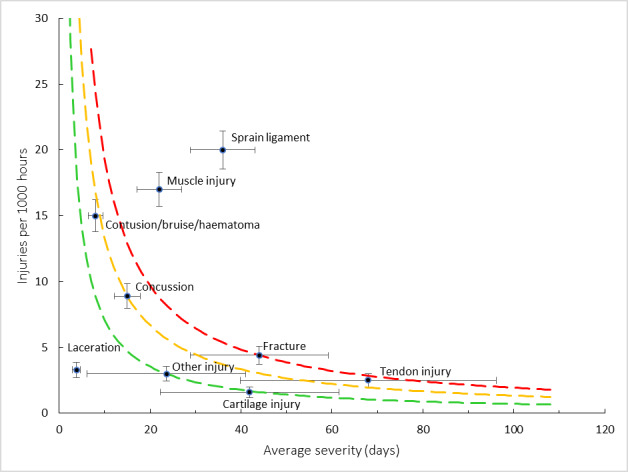
Injury burden as a function of injury type for the seasons 2016 – 2022. The y-axis represents incidence (number of injuries per 1000 hours), and x-axis represents the average severity (days absence) per injury type. Green line: values to the left and below represent those under the 25^th^ burden percentile; these are low-risk injuries. Orange line: values to the left and below represent those under the 50^th^ burden percentile; these include the low-medium risk injuries. Red line: values to the left and below represent those under the 75^th^ burden percentile; these include the medium-high risk injuries. Values to the right and above the red line are the most high-risk types of injuries, and impact players and teams the most.

Figure 9 shows the injury burden for the period 2016–2022. Ligament sprain followed by muscle injury were the two injury types with the highest burden when data were combined for the 2016–2022 Currie Cup tournaments. These injury types have the highest combination of injury incidence and average severity of injury. Consistent with previous reports, these two injury types continue to dominate across the different teams.

The most common Time-loss injuries during the Currie Cup 2022 tournament were joint (non-bone)/ligament injuries (comprised of dislocation/subluxation and sprain/ligament injuries) at 21 (14 to 28) injuries per 1000 player hours. The average severity of joint (non-bone)/ligament injuries in the Currie Cup 2022 was 38 (27 to 49) days.

Following joint (non-bone)/ligament injuries, muscle/tendon injuries (comprised of muscle rupture/strain/tear, tendon injury/rupture and tendinopathy injuries) were the next most common injury. The injury rate for muscle/tendon injuries was 17 (11 to 23) injuries per 1000 player hours. The average severity for muscle/tendon injuries was 36 (23 to 50) days. The injury incidence for the central nervous system during the Currie Cup 2022 was recorded at 10 (5 to 14) injuries per 1000 player hours. The average severity for central nervous system injuries was 24 (13 to 35) days.

### Injury Diagnosis [9]

The most common Orchard Sports Injury Classification System (OSIICS) diagnosis ^[10]^ in the Currie Cup 2022 was Concussion (OSIICS code = HNCX) followed by Hamstring Strain (TMHX) (Table 5).

## Concussions

**Figure 10:**
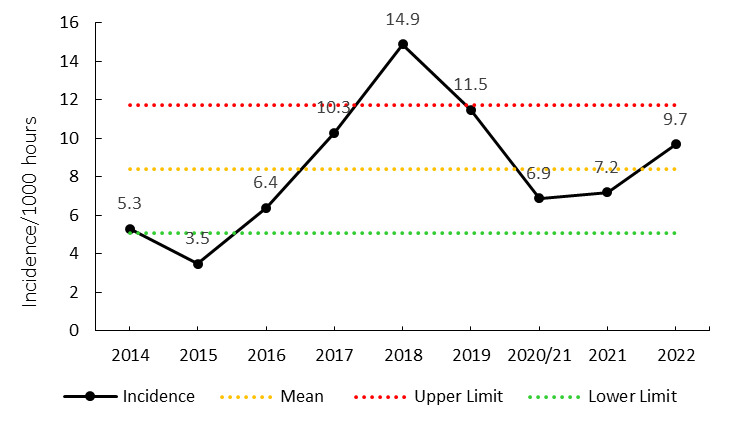
Incidence of concussion over the surveillance period with mean ± standard deviations shown. The red dotted line represents the mean plus standard deviation. The green dotted line represents the mean minus standard deviation. 2020/21 – was a hybrid tournament structure that started in 2020 and carried over into the beginning of the 2021 season due to Covid-19 lockdown interruptions.

Overall, concussions contributed to 17 injuries throughout the Currie Cup 2022 (14%). From 2020/21 to 2022, concussion rates have increased. Concussion incidence increased from 7.2 injuries per 1000 player hours in 2021 to 9.7 injuries per 1000 player hours in the Currie Cup 2022 tournament. This still falls within the expected season-to-season variation for the Currie Cup (Figure 10), with an overall grouped tournament average of 8.4 concussions per 1000 player hours over the data collection period. The average severity of concussions reported in the 2022 tournament was 24 days with a median of 13 days (IQR 12 – 27 days). The current South African Rugby concussion regulations do not normally allow for adult players to return to playing rugby within less than 12 days of the concussive event. Since this competition is held at the professional level and is a World Rugby approved tournament, the medical practitioners implement Advanced Care protocols. These protocols can potentially enable a player to return to play in less than 12 days. These regulations have recently been amended by World Rugby.

Advanced care clinical settings are defined in the World Rugby and SARU’s Concussion Guideline documents:

- World Rugby Concussion Guideline document: https://playerwelfare.worldrugby.org/
- SARU’s Concussion Guideline documents (When can a player safely return-to-play following a concussion): www.boksmart.com/concussion, and on *MyBokSmart*: https://my.boksmart.com/Documents/BokSmart#ConcussionManagement

**Figure 11:**
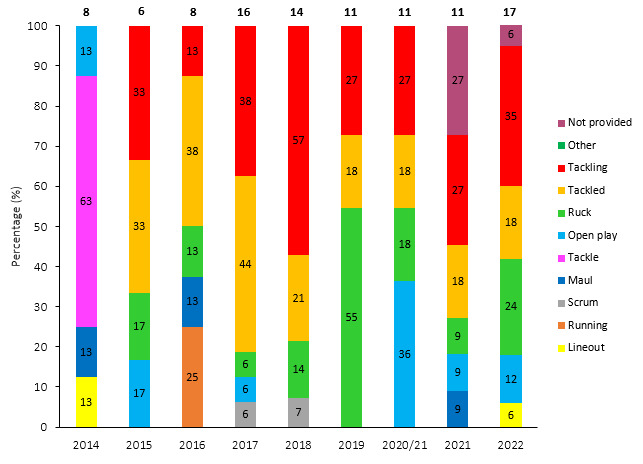
Proportion of concussions caused by the different injury events from 2014 to 2022. (The number above each bar represents the total number of concussions for that year). 2020/21 – was a hybrid tournament structure that started in 2020 and carried over into the beginning of the 2021 season due to Covid-19 lockdown interruptions.

Figure 11 shows the proportion of concussions caused by different injury events. Overall, the number of concussions increased since 2021. The main causes of concussion during the Currie Cup 2022 were *Tackling* (35%), followed by the *Ruck* (24%).

**Figure 12:**
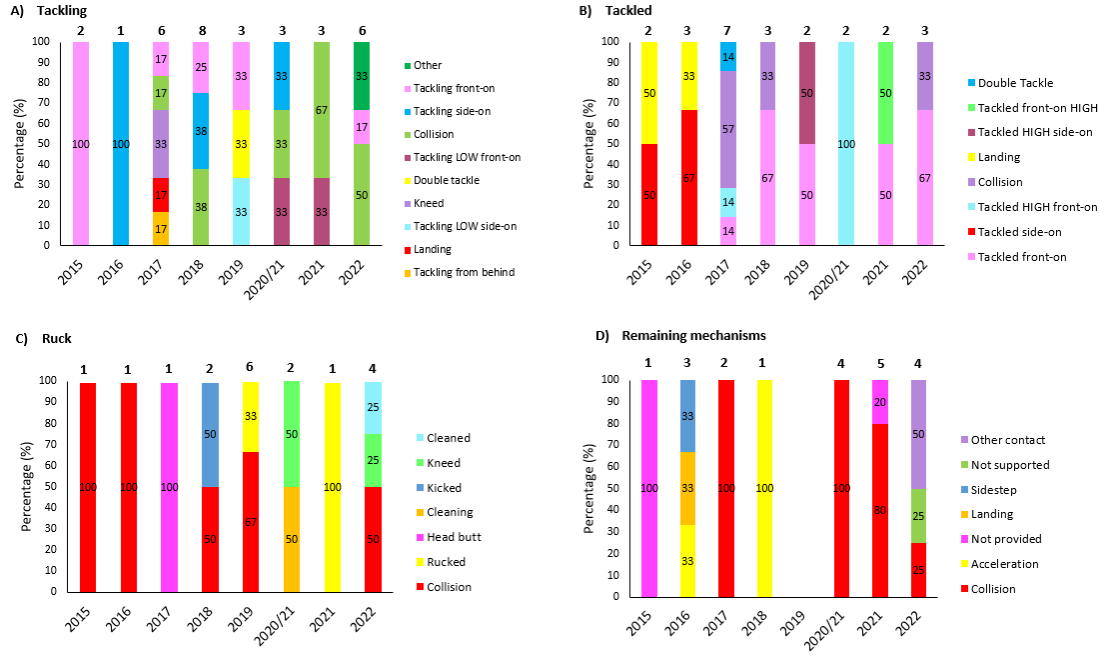
Proportion of concussions caused by A) Tackling, B) Tackled, C) Ruck and D) Remaining concussion mechanisms from 2015 to 2022. The number above each bar represents the total number of concussions for that event for that year. 2020/21 – was a hybrid tournament structure that started in 2020 and carried over into the beginning of the 2021 season due to Covid-19 lockdown interruptions.

Figure 12 presents the mechanisms contributing to concussions in *Tackling, Tackled, Ruck* and the remaining concussion causing injury events from 2015 – 2022. Data have only been presented from 2015 onwards as *Tackle* related data were not captured separately for the *Tackler* and *Ball Carrier* in 2014.

### Region of Injury

The shoulder and head were the most frequently injured body locations during the Currie Cup 2022 tournament (17% each), followed by the ankle (15%). Joint injuries (n = 6), followed by ligament injuries (n = 5), contributed to the most shoulder injuries. Concussions (n = 17) contributed to the most head injuries, whereas ligament injuries (n = 16) contributed to the most ankle injuries. Ligament injuries (n = 6) also accounted for most knee injuries. This was followed by joint injuries (n = 4), and muscle strain/spasm injuries (n ≤ 3). Muscle strain/spasm injuries (n = 14) contributed to the most thigh injuries.

The average severity for shoulder injuries was 55 days absent and injury burden 660 days absent per 1000 player hours: the highest injury severity and burden. This was followed by knee injuries with 46 and 533 days respectively. Head injuries had an average severity of 24 days absent, and an injury burden of 264 days absent per 1000 player hours. Thigh injuries had the lowest average severity of 29 days absent, and an injury burden of 198 days absent per 1000 player hours. The median severity of shoulder injuries was the highest in the Currie Cup 2022 at 31 days absent. Twenty-five percent of shoulder injuries resulted in 18 or less days lost from training and matches, and 25% of all shoulder injuries resulted in 73 or more days lost from training and matches (Table 6).

When analysing the changes in incidence of the most injured body locations for the Currie Cup over the past seven seasons (2016–2022), the head consistently ranks high on the list of the most frequently injured body locations. However, the shoulder has taken the top spot and increased by 6% since the 2021 season with knee injuries moving down the list (Table 7).

**Figure 13:**
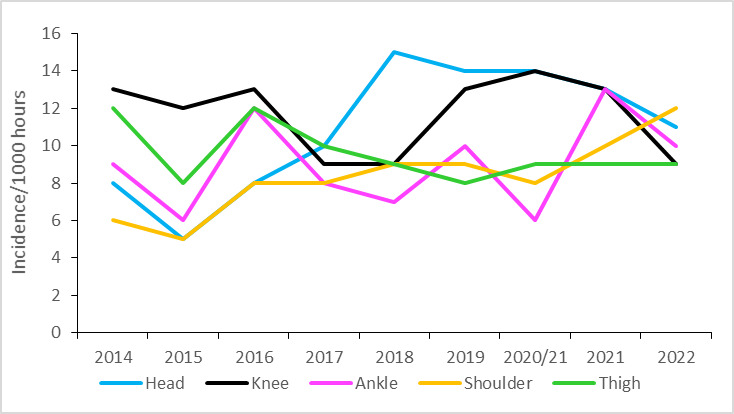
Incidence of the most common injury locations over the surveillance period. 2020/21 – was a hybrid tournament structure that started in 2020 and carried over into the beginning of the 2021 season due to Covid-19 lockdown interruptions.

Figure 13 displays the movement of the most common injured body locations over the surveillance period (2014–2022). When examining the injury incidence patterns over the past nine years, a clear upward trend can be observed in shoulder injuries since the 2020/21 season, with a gradual increase since 2015. In the 2022 season, shoulder injuries reached the highest level recorded in the past nine years. Thigh injuries have levelled out over the past three years. Head injuries increased initially from 2015 to 2018, after which they stabilised and are on a downward trend since then (Figure 13). The trend is not the same, but the head injury data here links directly to the concussion section earlier in the report, since most head injuries were attributed to concussions.

**Figure 14:**
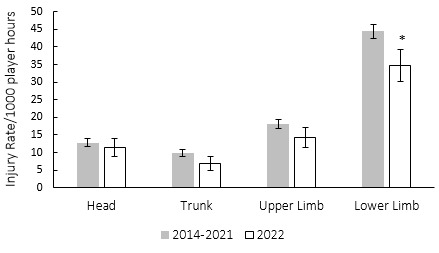
Injury incidence by grouped body location for the Currie Cup 2022 in comparison to the averaged 2014–2021 injury rates. The whiskers for each bar represent the 95% Confidence Intervals.

During the Currie Cup 2022, lower limb injury rates were significantly lower than their 2014–2021 averaged injury rates (Figure 14). During the Currie Cup 2022, the shoulder and head areas had the highest injury rates, with 12 (7 to 17) and 11 (6 to 16) injuries per 1000 player hours, respectively. The shoulder injury rate is similar to the meta-analysis [1] injury rate of 12 (10 to 14) injuries per 1000 player hours and the head injury rate was lower than that of the international meta-analysis [1] of 17 (14 to 20) injuries per 1000 player hours.

### Injury Event

The *Tackle (Ball Carrier)* event accounted for the most injuries in the Currie Cup 2022 (24%, n = 29), followed by *Open Play*, accounting for 18% of injuries (n = 22) (Table 8). When comparing injury rates to the international meta-analysis, *being tackled* at 17 (11 to 23) injuries per 1000 player hours during the Currie Cup 2022 was similar to the meta-analysis results of 23 (21 to 25) injuries per 1000 player hours. *Tackle (Tackler)* injury rate in the Currie Cup 2022 at 11 (6 to 16) injuries per 1000 player hours was significantly lower than the meta-analysis rate of 23 (21 to 25) injuries per 1000 player hours. Albeit less than *being tackled* in incidence, the average and median severity of injuries to the *tackler* were notably greater. *Ruck* injury rate during the 2022 season at 9 (4 to 13) injuries per 1000 player hours was similar to the meta-analysis injury rate of 9 (7 to 11) injuries per 1000 player hours [1].

**Figure 15:**
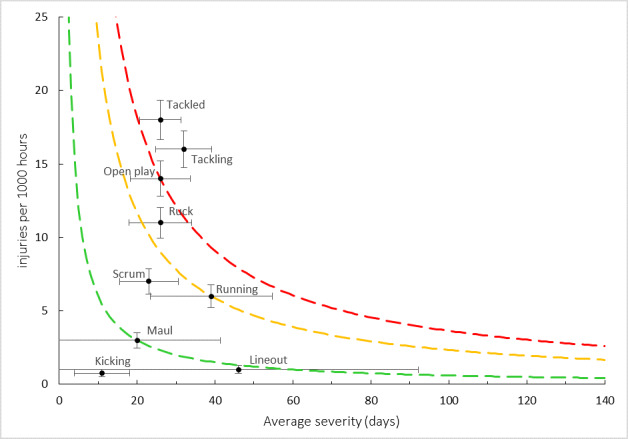
Injury burden as a function of injury event for the seasons 2016 – 2022. The y-axis represents incidence (injuries per 1000 hours), and x-axis represents average severity (days absence). Green line: values to the left and below represent those under the 25th burden percentile; these are low-risk injuries. Orange line: values to the left and below represent those under the 50th burden percentile; these include the low-medium risk injuries. Red line: values to the left and below represent those under the 75th burden percentile; these include the medium-high risk injuries. Values to the right and above the red line are the most high-risk types of injuries, and impact players and teams the most.

Figure 15 combines all the injury types from 2016 – 2022 and presents the injury burden picture over the past seven years. Injuries caused by *Tackling* have the highest injury burden for all teams, followed closely by injuries from *being tackled*. Both these injury events have a high combined injury incidence and average severity. *Open play* followed closely behind these two injury-causing events.

**Figure 16:**
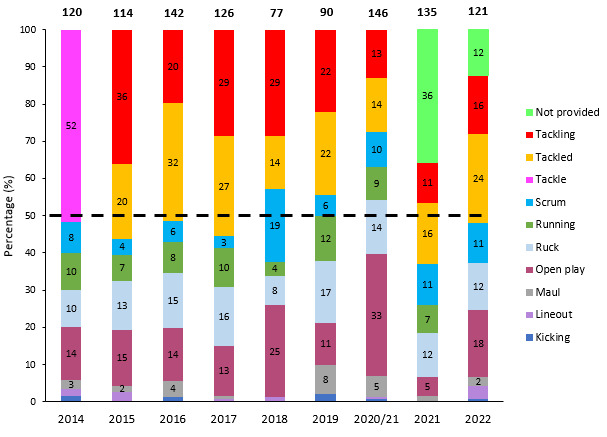
Proportion of injuries caused by the different injury events from 2014 to 2022. (The number above each bar represents the total number of injuries for that year. Tackle data captured separately as tackling and tackled from 2015 onwards). 2020/21 – was a hybrid tournament structure that started in 2020 and carried over into the beginning of the 2021 season due to Covid-19 lockdown interruptions.

Figure 16 illustrates the proportion of injuries caused by different injury events from 2014 to 2022. Over the past six seasons, the rate of injuries caused by tackles has varied. Notably, the proportion of tackling-related injuries has remained relatively lower over the last three years.

### Venue

**Figure 17:**
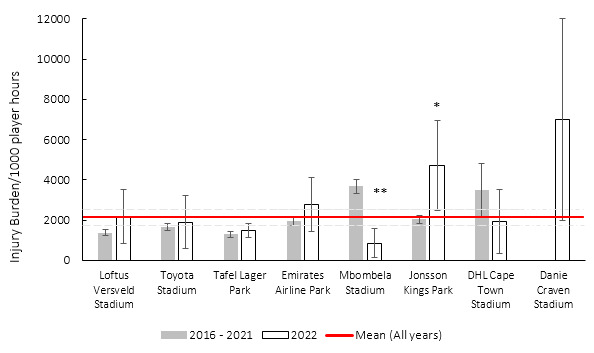
Injury burden/1000 player hours of Time-Loss injuries at the eight utilised stadia in the Currie Cup 2022 in comparison to their averaged 2016–2021 injury burden. *Stadium injury burden was significantly higher in 2022 than its 2016–2021 average. **Stadium injury burden was significantly lower in 2022 than its 2016–2021 average. The whiskers for each bar represent the 95% confidence intervals.

Matches were played at eight different stadia during the tournament. This is the first year that the Danie Craven Stadium was used during the Currie Cup tournament and was only used for one match. Danie Craven Stadium’s injury burden is far above the average injury burden and recorded the highest burden to date (Figure 17). Although with only one match played at the Danie Craven Stadium and the large 95%CI, one cannot draw any major conclusions. In 2022, Jonsson Kings Park’s injury burden was significantly higher than its 2016–2021 injury burden average, whereas Mbombela Stadium’s injury burden was significantly lower than its 2016–2021’s injury burden average.

Table 9 shows the ranking of injury burden of the Stadia from the highest to lowest between 2016–2022. When combining the last seven season’s data, it highlights that the Danie Craven Stadium, followed by Mbombela Stadium recorded the highest injury burdens overall, with only Mbombela’s averaged injury burden being significantly higher than the grouped average injury burden from 2016–2022 (Table 9).

**Figure 18:**
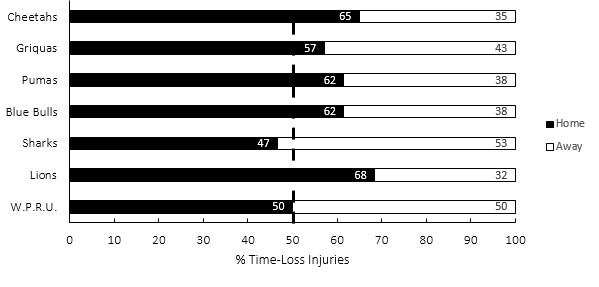
Proportion of injuries sustained playing at home and away venues for the Currie Cup 2022.

Figure 18 presents the proportion of injuries sustained playing at home and away venues in the Currie Cup 2022. When comparing injuries while playing away and at home in the Currie Cup 2022 tournament, playing at home at 40 (31 to 49) injuries per 1000 player hours, recorded a higher injury rate to playing away with 29 (21 to 37) injuries per 1000 player hours, but was not significantly different. With the exceptions of the Cell C Sharks and DHL Western Province (W.P.R.U.), all teams experienced more injuries when playing at home compared to playing away. DHL Western Province (W.P.R.U.) had an equal distribution of injuries at home and away matches, while Cell C Sharks experienced more injuries playing away.[Fig f28-2078-516x-35-v35i1a16880]

**Figure f28-2078-516x-35-v35i1a16880:**
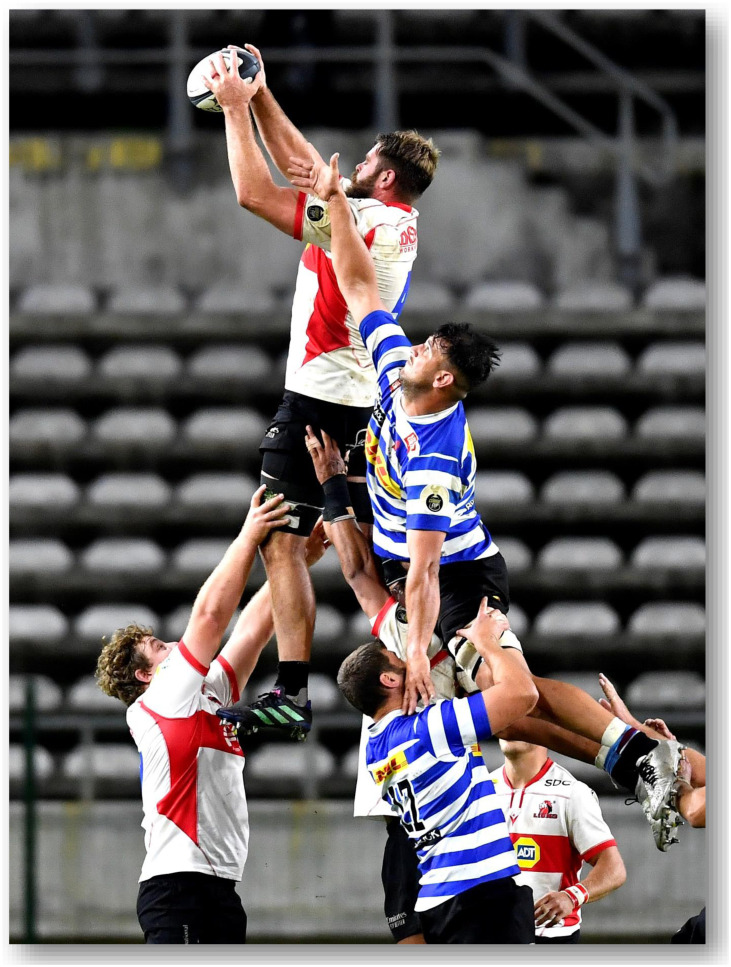


## TRAINING INJURIES

**Figure 19:**
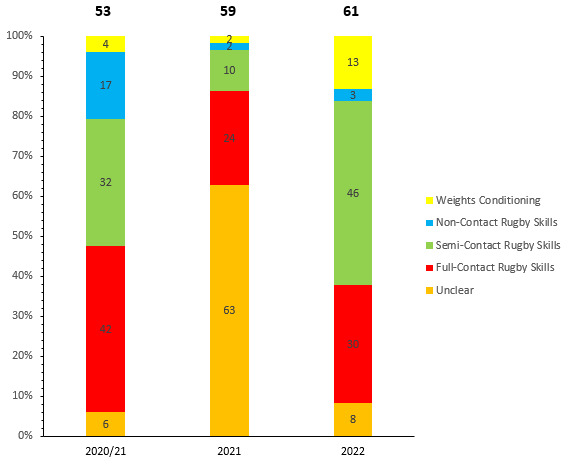
Proportion of Time-Loss training injuries sustained per training activity during the Currie Cup 2020/21–2022. 2020/21 – was a hybrid tournament structure that started in 2020 and carried over into the beginning of the 2021 season due to Covid-19 lockdown interruptions.

Overall, 61 Time-loss injuries were sustained during training in the Currie Cup 2022. The time-loss injuries resulted in an injury incidence of 2 (1.5 to 2.5) injuries per 1000 training hours which is lower than the meta-analysis injury incidence of 3 (1.9 to 4.0) injuries per 1000 training hours [1]. These time-loss injuries contributed to 34% of all injuries experienced during the Currie Cup Tournament over the 2022 rugby season. The average severity of training injury was 34 days, with a median severity (IQR) of 17 (10 to 46) days absent. Figure 19 shows the percentage of training injuries per training activity. Semi and full contact rugby skills accounted for the highest percentage of training injuries, which is expected given the nature of contact involved in those activities. Injuries associated with weights conditioning training increased (Table 10).

Table 10 presents the training injuries sustained during the Currie Cup 2022. The most common injury type sustained in *full contact* rugby skill activities was *Muscle Injuries*, and *Joint Injuries* had the highest average severity at 136 days (Table 10). Within *semi-contact* rugby skills, *Ligament Sprains* were the most common, whereas *Joint Injuries* again had the highest average severity of 143 days.

The *thigh* was the most injured body location in training accounting for 18% (n = 11) of all Time-Loss training injuries during the Currie Cup 2022, followed closely by the *ankle* (15%) and *head* (13%) (Table 11). *Hip/Groin* training injuries clearly had the highest average and median severities, followed by *shoulder* training injuries.[Fig f29-2078-516x-35-v35i1a16880]

**Figure f29-2078-516x-35-v35i1a16880:**
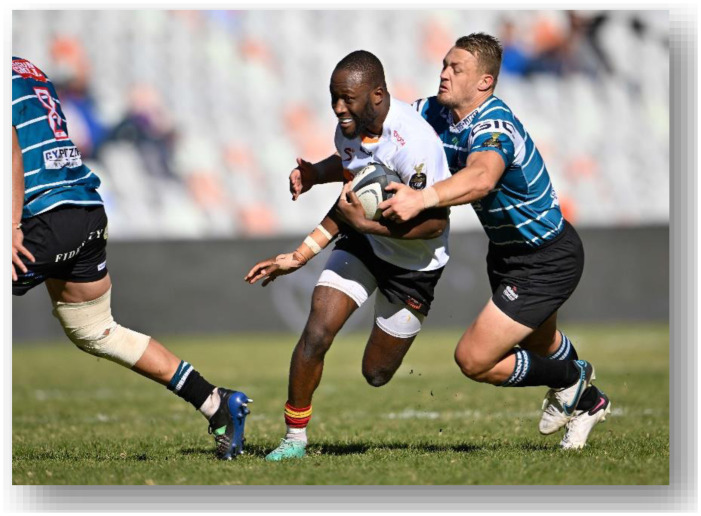


**Figure 20:**
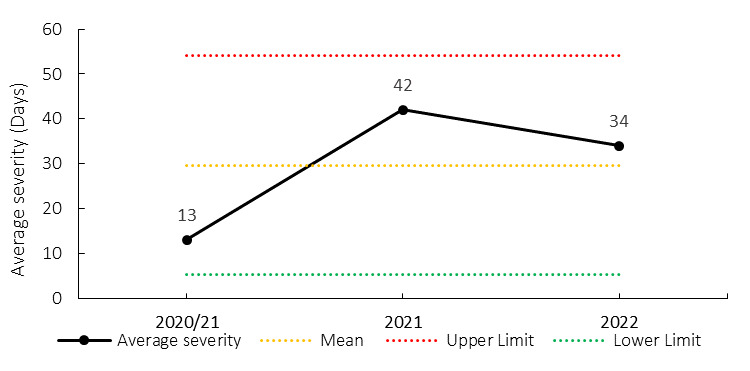
Mean severity of Time-Loss training injuries over the surveillance period with mean ± standard deviations shown. 2020/21 – was a hybrid tournament structure that started in 2020 and carried over into the beginning of the 2021 season due to Covid-19 lockdown interruptions.

The average severity of training injuries for the Currie Cup 2022 was 34 days, which is higher than the average severity for 2020/21 but lower than the 2021 Currie Cup tournament average (42 days) (Figure 20).

Overall, concussions contributed to 3 training injuries throughout the Currie Cup 2022 (3%). This is similar to the 2020/21 and 2021 Currie Cup seasons.[Fig f30-2078-516x-35-v35i1a16880]

**Figure f30-2078-516x-35-v35i1a16880:**
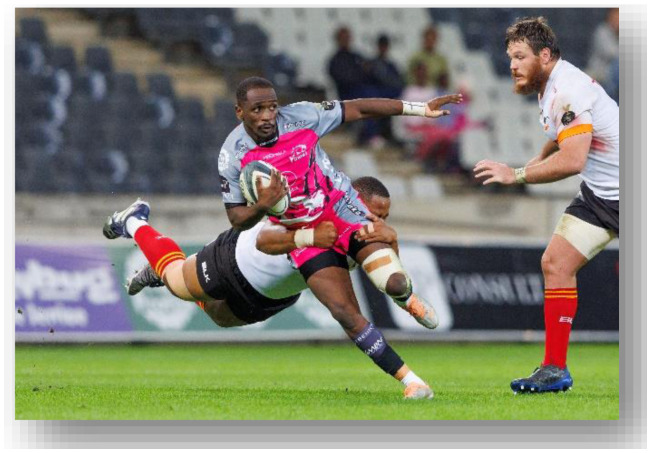


## Figures and Tables

**Figure 1a:**
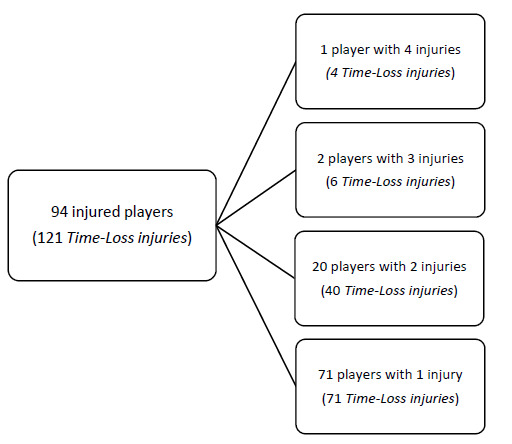
The number of players who experienced Time-Loss injuries during the Currie Cup 2022.

**Figure 1b:**
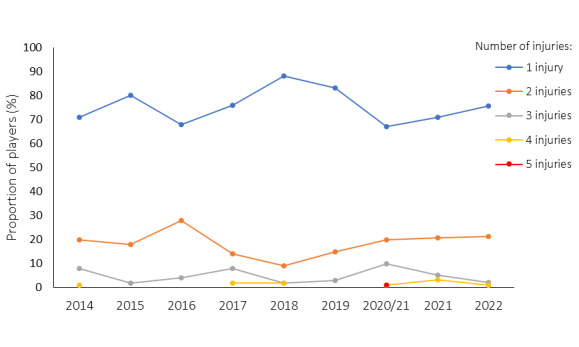
Proportion of injured players experiencing one to five injuries in the Currie Cup tournaments from 2014–2022. 2020/21 – was a hybrid tournament structure that started in 2020 and carried over into the beginning of the 2021 season due to Covid-19 lockdown interruptions.

**Figure 21:**
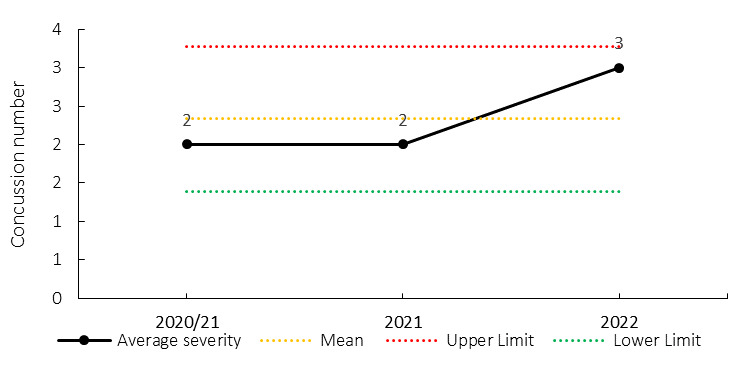
Absolute number of concussions recorded over the training surveillance period. 2020/21 – was a hybrid tournament structure that started in 2020 and carried over into the beginning of the 2021 season due to Covid-19 lockdown interruptions.

**Table 1:** Injury Incidence, Severity (days), Injury Burden (days absent/1000 player hours) and Operational Burden (days absent/injury/match) of Time-Loss injuries for each participating team in the Currie Cup 2022.

Team	Team Injuries / match	Injury Incidence (per 1000 player hours)	Team matches / injury	Total Severity (days lost)	Average Severity (days lost/injury)	Injury Burden (days lost / 1000 hours)	Operational Injury Burden (days lost / injury / match)	Median Severity (IQR)
**Airlink Pumas**	0.9	46.4	1.1	163	13	582	11.6	11 (10 to 27)
**Vodacom Blue Bulls**	1.1	54.2	0.9	653	50	2723	54.5	20 (9 to 57)
**Toyota Free State Cheetahs**	1.5	76.9	0.7	372	19	1430	28.6	12 (9 to 17)
**Sigma Golden Lions**	1.6	79.2	0.6	753	40	3139	62.8	37 (13 to 58)
**Cell C Sharks**	2.7	136.4	0.4	951	32	4324	86.5	27 (17 to 44)
**DHL Western Province**	1.0	50.0	1.0	345	29	1438	28.8	10 (5 to 52)
**Windhoek Draught Griquas**	1.0	50.0	1.0	530	38	1893	37.9	24 (15 to 65)
***Overall***	***1.4***	***68.8***	***0.7***	***3737***	***31***	***2139***	***42.8***	***19 (10 to 44)***

**Table 2:** The Currie Cup 2022 injuries grouped according to the IOC recommended categories of Tissue and Pathology types for injuries.

Tissue	Incidence	Median time loss	Burden
*Pathology*	Injuries per 1000 hours (95%CI)	Days (95%CI)	*Days per 1000 hours (95%CI)*

**Muscle/Tendon**	**17 (11 to 23)**	**27 (6 to 48)**	**765 (495 to 1035)**
Muscle Injury	14 (9 to 20)	23 (13 to 33)	420 (210 to 600)
Tendon rupture	1 (1 to 2)	270	270
Tendinopathy	2 (0 to 4)	90 (0 to 182)	178 (41 to 356)
**Ligament/Joint capsule**	**24 (17 to 32)**	**25 (13 to 37)**	**1008 (714 to 1344)**
Ligament Sprain	18 (11 to 24)	24 (12 to 36)	756 (462 to 1008)
Joint Sprain	7 (3 to 11)	26 (0 to 58)	294 (126 to 462)
**Nervous**	**11 (6 to 16)**	**13 (3 to 23)**	**275 (150 to 400)**
Brain/Spinal cord injury	10 (5 to 14)	13 (2 to 24)	240 (120 to 336)
Peripheral nerve injury	1 (0 to 3)	28 (0 to 65)	28 (0 to 84)
**Superficial tissues/skin**	**8 (4 to 12)**	**10 (6 to 14)**	**88 (44 to 132)**
Contusion (superficial)	8 (4 to 12)	10 (6 to 14)	88 (44 to 132)
**Bone**	**4 (1 to 7)**	**40 (22 to 58)**	**148 (37 to 259)**
Fracture	4 (1 to 7)	40 (22 to 58)	148 (37 to 259)
**Cartilage/Synovium/Bursa**	**2 (0 to 4)**	**126 (92 to 160)**	**252 (0 to 504)**
Bursitis	1 (0 to 2)	205	205
Cartilage Injury	1 (0 to 3)	147	147
**Non-specific**	**3 (1 to 6)**	15 (5 to 25)	48 (16 to 96)

***Overall***	***69 (57 to 81)***	***19 (10 to 44)***	***2139 (1767 to 2511)***

**Table 3:** Proportion (%) of new versus subsequent recurrent injuries for the Currie Cup 2016 – 2022 tournaments.

	2016	2017	2018	2019	2020/21	2021	2022
New injuries	74	74	86	83	68	71	78
Subsequent recurrent injuries	2.8	3.2	2.6	2.2	3.4	4.4	3.3

**Table 4:** Injury rate, Severity and Burden of the most common injury types in the Currie Cup 2022.

Injury Type	Injury Rate (*95%* CI) *(per 1000 hours)*	Total Severity *(days)*	Average Severity *(days)*	Burden (*95%* CI) *(days lost / 1000 hours)*	Median (IQR)
Sprain Ligament	18 (11 to 24)	968	42	756 (462 to 1008)	24 (13 to 75)
Muscle (Rupture/Strain/Tear)	14 (9 to 20)	754	30	420 (210 to 600)	23 (12 to 38)
Central Nervous System	10 (5 to 14)	412	24	240 (120 to 336)	13 (12 to 27)
Contusion/Bruise	8 (4 to 12)	149	11	88 (44 to 132)	10 (6 to 13)
Tendons	2 (0 to 5)	537	134	268 (0 to 670)	130 (69 to 195)

***Overall***	***69 (57 to 81)***	***3737***	***31***	***2139 (1767 to 2511)***	***19 (10 to 44)***

**Table 5:** The movement of the most common OSIICS classification diagnoses over the past seven seasons [9]. 2020/21 – was a hybrid tournament structure that started in 2020 and carried over into the beginning of the 2021 season due to Covid-19 lockdown interruptions.

			Percentage (%)	Number	Incidence (*95%* CI)	Average Severity
2016		Concussion (HN1)	7	10	6 (2–10)	14
		Knee medial collateral ligstr/tear/rupture (KL3)	6	9	6 (2–10)	23
		Hamstring strain/tear (TM1)	6	8	5 (2–9)	11
2017		Concussion (HNCX)	13	16	10 (5–15)	15
		Acromioclavicular jt sprain (SJAX)	10	12	8 (3–12)	25
2018		Concussion (HNCX)	18	14	15 (7–23)	14
		Quadricep strain (TMQX)	5	4	4 (0–8)	18
2019		Concussion (HNCX)	12	11	12 (5–18)	9
		Ankle syndesmosis sprain (AJSX)	5	5	5 (1–10)	14
2020/21		Concussion (HNCX)	8	11	7 (3–11)	10
		Quadriceps haematoma (THV)	4	6	4 (1–7)	4
		Knee strain (MCL)	3	5	3 (1–6)	42
2021		Concussion (HNCX)	8	11	7 (3–12)	15
		Ankle sprain (AJXX)	6	8	6 (2–10)	10
		Hamstring strain (THHX)	2	5	2 (0–4)	23
2022		Concussion (HNCX)	14	17	10 (5–14)	24
		Hamstring strain (TMHX)	6	7	4 (1–7)	46
		Ankle syndesmosis sprain (AJSX)	4	5	3 (0–5)	12

**Table 6:** Injury rate, Severity and Burden of the most common injury types in the Currie Cup 2022.

Injury type	Injury Rate (*95%* CI) *(per 1000 hours)*	Total Severity *(days)*	Average Severity *(days)*	Burden (*95%* CI) *(days lost / 1000 hours)*	Median (IQR)
Shoulder	12 (7 to 17)	823	55	660 (385 to 935)	31 (18 to 73)
Head	11 (6 to 16)	488	24	264 (144 to 384)	13 (12 to 31)
Ankle	10 (5 to 15)	392	30	300 (150 to 450)	16 (9 to 50)
Knee	9 (5 to 14)	507	46	533 (287 to 779)	30 (12 to 75)
Thigh Injuries	9 (5 to 14)	469	29	198 (88 to 308)	22 (10 to 40)

***Overall***	***69 (57 to 81)***	***3737***	***31***	***2139 (1767 to 2511)***	***19 (10 to 44)***

**Table 7:** The movement of the most injured body locations over the past seven seasons. 2020/21 – was a hybrid tournament structure that started in 2020 and carried over into the beginning of the 2021 season due to Covid-19 lockdown interruptions.

			Percentage (%)	Number	Incidence (95% CI)	Average Severity
2016		Knee	14	20	13 (7–18)	49
		Ankle	13	18	12 (6–17)	51
		Head	9	13	8 (4–13)	11
		Shoulder	8	12	8 (3–12)	41
2017		Head	13	16	10 (5–15)	15
		Knee	11	14	9 (4–14)	63
		Shoulder	10	12	8 (3–12)	67
		Ankle	10	12	8 (3–12)	87
		A/C Joint	10	12	8 (3–12)	25
2018		Head	18	14	15 (7–23)	18
		Knee	10	8	9 (3–14)	44
		Shoulder	10	8	9 (3–14)	38
		Ankle	9	7	7 (2–13)	65
		Anterior thigh	8	6	6 (1–12)	6
2019		Head	14	13	14 (6 – 21)	8
		Knee	13	12	13 (5 – 20)	13
		Ankle	11	10	10 (4 – 17)	9
		Lower limb posterior	7	6	6 (1 – 11)	3
		Posterior thigh	7	6	6 (1 – 11)	9
2020/21		Head	16	23	14 (9 to 20)	6
		Knee	15	22	14 (8 to 19)	57
		Thigh	10	15	9 (5 to 14)	9
		Shoulder	9	13	8 (4 to 13)	22
		Ankle	7	10	6 (2 to 10)	19
2021		Head	15	20	13 (7 to 19)	9
		Knee	15	20	13 (7 to 19)	41
		Ankle	14	19	13 (7 to 18)	13
		Shoulder	11	15	10 (5 to 15)	18
		Thigh	10	14	9 (4 to 14)	22
2022		Shoulder	17	21	12 (7 to 17)	55
		Head	17	20	11 (6 to 16)	24
		Ankle	15	18	10 (5 to 15)	30
		Knee	13	16	9 (5 to 14)	46
		Thigh	13	16	9 (5 to 14)	29

**Table 8:** Injury rate, Severity and Burden of the injury events in the Currie Cup 2022

Injury event	Injury Rate (*95%* CI) *(per 1000 hours)*	Total Severity *(days)*	Average Severity *(days)*	Burden (*95%* CI) *(days lost / 1000 hours)*	Median (IQR)
Tackle (Ball Carrier)	17 (11 to 23)	750	36	612 (396 to 828)	17 (10 to 50)
Open play	13 (7 to 18)	666	37	481 (259 to 66)	26 (18 to 59)
Tackle (Tackler)	11 (6 to 16)	835	49	539 (294 to 784)	31 (13 to 45)
Ruck	9 (4 to 13)	284	20	180 (80 to 260)	12 (10 to 25)
Scrum	7 (3 to 11)	341	28	196 (84 to 308)	14 (9 to 30)
Lineout	2 (0 to 5)	66	22	44 (0 to 110)	13 (10 to 30)
Maul	2 (0 to 4)	46	15	30 (0 to 60)	11 (8 to 21)
Kicking	1 (0 to 2)	5	5	5	5
Other	9 (4 to 13)	299	25	225 (100 to 325)	16 (10 to 30)
***Overall***	***69 (57 to 81)***	***3737***	***31***	***2139 (1767 to 2511)***	***19 (10 to 44)***

**Table 9:** Injury burden/1000 hours of Time-Loss injuries at the eight Stadia utilized in the Currie Cup combined data from 2016 to 2022.

*Stadium*	*Burden (95%Cl)*
*Danie Craven Stadium*	*7000 (140 to 13860)*
***Mbombela Stadium***	***3167 (2575 to 3758)***
*DHL Cape Town Stadium*	*2464 (1457 to 3471)*
*Jonsson Kings Park*	*2429 (2020 to 2837)*
*Emirates Airline Park*	*2079 (1674 to 2485)*
*Toyota Stadium*	*1727 (1424 to 2030)*
*Loftus Versveld Stadium*	*1462 (1193 to 1730)*
*Tafel Lager Park*	*1214 (956 to 1472)*

***Grouped Average***	***2128 (1741 to 2517)***

**Table 10:** Injury incidence rate, average-, and median severity of training injuries sustained during the Currie Cup 2022 season according to type of training activity involved.

	Injury Incidence (per 1000 player hours)	Average severity (days)	Median severity (days)
***Rugby skills (full contact)***	***0.6 (0.3 to 0.6)***	***34***	***17***
Muscle injury	0.2 (0.0 to 0.4)	18	16
Ligament sprain	0.1 (0.0 to 0.3)	12	7
Concussion	0.1 (0.0 to 0.2)	16	16
Joint injury	0.1 (0.0 to 0.2)	136	136
Tendon injury	0.1 (0.0 to 0.2)	96	96
Fracture	0.0 (0.0 to 0.1)	31	31
***Rugby skills (semi-contact)***	***0.9 (0.6 to 1.3)***	***35***	***21***
Ligament sprain	0.3 (0.1 to 0.4)	37	20
Muscle injury	0.1 (0.0 to 0.3)	29	28
Fracture	0.1 (0.0 to 0.3)	58	56
Concussion	0.1 (0.0 to 0.3)	13	11
Tendon injury	0.1 (0.0 to 0.2)	15	15
Bruising/ haematoma	0.1 (0.0 to 0.2)	21	21
Joint injury	0.0 (0.0 to 0.1)	143	143
Synovitis/capsulitis	0.0 (0.0 to 0.1)	10	10
Laceration	0.0 (0.0 to 0.1)	7	7
***Rugby skills (non-contact)***	***0.1 (0.0 to 0.2)***	***8***	***6***
Muscle injury	0.0 (0.0 to 0.1)	8	8
Ligament sprain	0.0 (0.0 to 0.1)	6	6
***Weights Conditioning***	***0.3 (0.1 to 0.4)***	***51***	***56***
Muscle injury	0.1 (0.0 to 0.2)	45	45
Synovitis/capsulitis	0.1 (0.0 to 0.2)	47	47
Nerve injury	0.0 (0.0 to 0.1)	66	66
Joint injury	0.0 (0.0 to 0.1)	56	56
Tendon injury	0.0 (0.0 to 0.1)	14	14
Non-specific	0.0 (0.0 to 0.1)	88	88
***Other***	***0.2 (0.0 to 0.3)***	***9***	***8***
Muscle injury	0.1 (0.0 to 0.2)	10	11
Ligament sprain	0.0 (0.0 to 0.1)	7	7
Tendon injury	0.0 (0.0 to 0.1)	7	7
***Overall***	***2.0 (1.5 to 2.5)***	***34***	***17***

**Table 11:** Injury incidence rate, average-, and median severity of training injuries sustained per body location, during the Currie Cup 2022.

	Injury Incidence (per 1000 player hours)	Average severity (days)	Median severity (days)
***Head***	**0.3 (0.1 to 0.4)**	**13**	**12**
***Neck***	0.1 (0.0 to 0.2)	**9**	**11**
***Upper Body***	***0.4 (0.2 to 0.6)***	***51***	***27***
Shoulder	0.2 (0.1 to 0.4)	72	71
Wrist/hand	0.1 (0.0 to 0.3)	35	30
Elbow	0.0 (0.0 to 0.1)	27	27
***Lower Body***	***1.1 (0.7 to 1.4)***	***36***	***22***
Thigh	0.4 (0.1 to 0.6)	27	28
Ankle	0.3 (0.1 to 0.5)	31	14
Knee	0.2 (0.0 to 0.4)	45	45
Foot	0.1 (0.0 to 0.2)	34	34
Hip/groin	0.1 (0.0 to 0.2)	131	131
Lower leg	0.1 (0.0 to 0.2)	9	8
***Trunk***	***0.3 (0.1 to 0.4)***	***33***	***31***
Chest	0.1 (0.0 to 0.2)	39	39
Lumbar spine	0.1 (0.0 to 0.2)	30	17
***Overall***	***2.0 (1.5 to 2.5)***	***34***	***17***
